# Trained immunity in skin infections: Macrophages and beyond

**DOI:** 10.7554/eLife.106688

**Published:** 2025-10-02

**Authors:** Vitka Gres, Merve Göcer, Julia Kolter, Philipp Henneke

**Affiliations:** 1 Institute for Infection Prevention and Control, Medical Center and Faculty of Medicine, University of Freiburg Freiburg Germany; 2 Center for Chronic Immunodeficiency, Medical Center and Faculty of Medicine, University of Freiburg Freiburg Germany; 3 Faculty of Biology, University of Freiburg Freiburg Germany; 4 Institute for Immunodeficiency, Medical Center and Faculty of Medicine, University of Freiburg Freiburg Germany; Murdoch Childrens Research Institute Australia; Indian Institute of Science Education and Research (IISER) India

**Keywords:** trained immunity, skin, macrophages, *S. aureus*, Dermis

## Abstract

The skin is frequently subjected to minor mechanical insults that may compromise its barrier integrity and permit the entry of pathogens. Therefore, the immune system of the skin needs to rapidly balance antimicrobial defense with tissue repair. To maintain homeostasis, the skin relies both on acute immune defenses and on mechanisms of innate memory or trained immunity. This enhanced inflammatory response to a second challenge has been well characterized in bone marrow cells, such as monocytes, monocyte-derived macrophages, and stem cells. Yet, the specific memory responses in skin-resident immune cells remain less understood. Importantly, the common skin colonizer *Staphylococcus aureus* has been identified as a potent inducer of trained immunity, triggering both metabolic and epigenetic changes at local sites such as the skin, and centrally in the bone marrow. This review explores the emerging understanding of trained immunity in the skin, that is how infection-driven cellular processes induce long-lasting immune adaptation and modulate skin barrier integrity.

## Immune landscape of the skin

The skin consists of two basic layers: the epidermis and the dermis. The epidermis forms the outermost layer, and it is separated from the underlying dermis by a basement membrane. It provides protection against pathogens and UV light, while preventing excessive water loss from the inside. The dermis contributes to the mechanical properties of the skin, such as elasticity and flexibility, due to its high content of collagen and elastin fibers. It also contains various appendages, including sweat and sebaceous glands and hair follicles, which contribute to physical protection, thermoregulation, sensation, and lubrication of the skin.

Below the dermis is the subcutis or hypodermis, which consists mainly of adipocytes and connective tissue and connects the skin to the deep fascia. The blood vessels in the subcutis feed into the deep venous plexus of the dermis, where they connect with the venules and arterioles of the superficial plexus, providing nutrients and oxygen to the epidermis and skin appendages.

### Epidermis

Keratinocytes are the most numerous cell type in the epidermis, ensuring its physical stability. Beyond this structural role, they also sense microbial components via Toll-like receptors (TLR), NOD-like receptors (NLRs), and C-type lectin receptors like dectin-1, leading to the production of cytokines, such as IL-1β and IL-36. These are important for the antimicrobial and immunological properties of the skin (Goldstein et al., 2020; Klicznik et al., 2018; Pfaff et al., 2017). Keratinocytes closely interact with Langerhans cells (LCs), a unique type of antigen-presenting cells. As an example, the maintenance and renewal of LCs in steady state relies on IL-34 produced by keratinocytes (Greter et al., 2012). Although LCs share many characteristics with dendritic cells (DCs), they are considered to be long-lived, self-renewing resident macrophages (Mϕ; Doebel et al., 2017; Mass et al., 2016). They derive from embryonic precursors, which transmigrate from the dermis and differentiate postnatally into a mature, self-renewing network of interdigitating cells (Hoeffel et al., 2012). However, upon inflammation or injury, ‘short-term’ monocyte-derived LCs can infiltrate the niche (Merad et al., 2008; Seré et al., 2012).

The lower epidermal layer in mice, the stratum spinosum, hosts a distinct population of branched γẟ T-cells, known as dendritic epidermal T-cells (DETC). They sense stress or damage in keratinocytes, aiding in wound healing and skin homeostasis. In humans, most epidermal T-cells are αβ T-cells, particularly a non-circulating resident memory population, seeding the skin following the resolution of infection and contributing to the long-term tissue surveillance (Ho and Kupper, 2019).

### Dermis and dermal macrophages

Unlike the epidermis, the dermis is populated by diverse myeloid and lymphoid immune cell populations (Kabashima et al., 2019). The healthy dermis is inhabited by innate lymphoid cells (ILCs), in particular ILC2, and different T-cell subsets, that is dermal γẟ T-cells, mucosa-associated invariant T-cells (MAIT), and CD4 + cells (Ho and Kupper, 2019). The dermis also supports mast cells, eosinophils, and numerous DC populations, with conventional CD11b^+^ DCs being the most prevalent (Belkaid and Tamoutounour, 2016).

In terms of absolute numbers, Mϕ are the dominant immune cell type in the dermis. Dermal Mϕ are a heterogeneous population in the adult, consisting of multiple subpopulations with distinct lifespans, localizations, and transcriptional profiles (Tamoutounour et al., 2013, Kolter et al., 2019; Lohrmann et al., 2025). As most tissues, the dermis is initially seeded by erythro-myeloid progenitors derived from the yolk sac and later the fetal liver (Gomez Perdiguero et al., 2015). While these embryonic Mϕ proliferate locally and persist into adulthood, they can also be replaced to tissue-specific extents by monocyte-derived Mϕ derived from hematopoietic stem cells in the bone marrow. In the dermis, Mϕ receive a significant input by circulating monocytes, leading to replacement of up to 50% of the population (Kolter et al., 2019; Lohrmann et al., 2025). Once in the tissue, their functions diversify depending on neighboring skin structures and the duration of their exposure to the microenvironment (Lohrmann et al., 2025). For example, Mϕ with long-term residency have a more immunosuppressive phenotype, akin to LCs in the epidermis, while infiltrating monocyte-derived Mϕ are more primed towards immune response and inflammation (Lohrmann et al., 2025). Moreover, LYVE-1^+^ dermal Mϕ associated with blood vessels have distinct features and functions compared to, for example, long-lived CX_3_CR1^hi^ sensory nerve-associated Mϕ, which are differentially imprinted by their TGF-β-rich environment (Chakarov et al., 2019; Kolter et al., 2019; Kolter et al., 2025).

Interestingly, the density of the resident Mϕ network in the skin is strictly regulated at the site, as demonstrated in Irf8^-/-^ mice, which exhibit a complete lack of circulating monocytes, yet show the same Mϕ density and diversity of transcriptional states as wild-type mice (Lohrmann et al., 2025).

In response to infection or injury, Mϕ are essential for maintaining skin barrier integrity and repair (Barreiro et al., 2016; Kolter et al., 2019; Shook et al., 2016). However, infections often lead to depletion of the resident Mϕ pool, followed by a replacement with infiltrating monocyte-derived Mϕ (Forde et al., 2023; Ginhoux et al., 2017). This dynamic turnover has important implications, as both cellular longevity and systemic cues may influence the development and maintenance of trained immunity, which we will explore in the following section.

## Trained immunity in the skin

### The concept of trained immunity

Trained immunity (TI) or innate immune memory is a process by which innate immune cells, which previously encountered a pathogenic stimulus, show an enhanced and more rapid response to a subsequent pathogenic challenge. This primed response pattern is characterized by enhanced formation of cytokines, reactive oxygen species (ROS), and increased phagocytosis (Netea et al., 2011). The concept of immune memory was traditionally associated with only adaptive immune cells (T- and B-cells) responding to repeated exposure to an identical molecular motif. However, the inducible protection against reinfection in plants and invertebrates, which lack adaptive immune cells, has led to questioning this paradigm and exploring the capacities of innate immune cells (Janeway, 1989). TI can last from weeks to several years, but it is generally shorter than adaptive immune memory (Netea et al., 2020).

While the tuberculosis vaccine Bacillus Calmette-Guérin (BCG) and the fungal cell wall component β-glucan were most frequently used as experimental inducers of TI, other triggers have been identified, for example oxidized low-density lipoproteins and *S. aureus* (Bekkering et al., 2014; Carlile et al., 2024; Feuerstein et al., 2020; Kaufmann et al., 2018; Moorlag et al., 2020). Consistent with the diversity of microbial triggers, the observed TI responses have been found to be quite variable in terms of cell or tissue type and altered effector patterns, including primary and secondary responses against the same pathogens or their effector molecules (i.e. homologous TI) or between different pathogens (heterologous TI).

**Figure 1.**
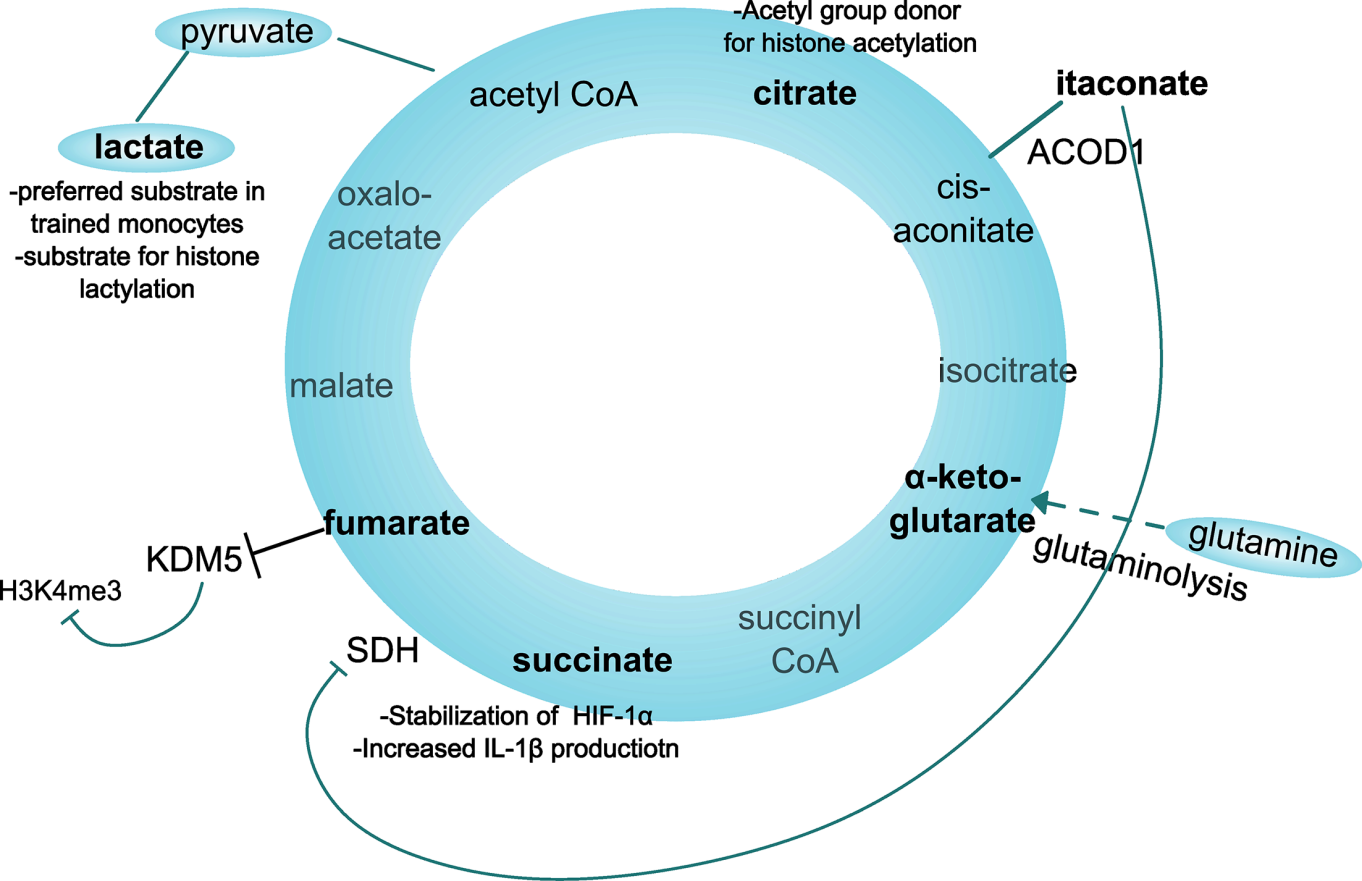
*Metabolites of the TCA cycle involved in trained immunity*. ACOD1 - aconitate decarboxylase, encoded by Immune responsive gene 1 (IRG1). SDH – succinate dehydrogenase, KDM5 - Lysine-specific demethylase 5.

One important example of heterologous TI is the protective effect of BCG vaccination against *Candida albicans* in severe combined immunodeficient (SCID) mice (Kleinnijenhuis et al., 2012; van ’t van ’t Wout et al., 1992), which lack functional T- and B- cells, highlighting the critical role of innate immune cells in memory responses. The initiation and maintenance of innate memory involves a complex interplay between epigenetic modifications and metabolic reprograming. For example, in infections, high glycolytic activity and byproducts of the tricarboxylic acid (TCA) cycle (e.g. fumarate) can modulate enzymes which mediate histone modification, thereby altering chromatin accessibility and the transcription of inflammatory genes (Figure 1; Arts et al., 2016; Riksen and Netea, 2021).

### Innate immune responses to *S. aureus*

To date, much of the information regarding TI in the skin is derived from studies using *S. aureus* as a TI inducer. However, the skin is frequently exposed to a wide range of viral, bacterial, and fungal pathogens, including herpes simplex virus (HSV), *Streptococcus pyogenes, C. albicans,* and others. Despite their clinical significance, studies into how these pathogens trigger TI in the skin remain very limited. Consequently, this review will primarily focus on insights gained from *S. aureus*-based models.

*S. aureus* is a skin colonizer in around 30% of humans (Eriksen et al., 1995; Kluytmans and Wertheim, 2005)*.* However, barrier disruption or immune dysregulation can enable opportunistic pathogenicity, making *S. aureus* the leading cause of skin and skin structure infections. *S. aureus* uses a multitude of means to evade host immunity, e.g., via inhibition of phagocytosis, degradation of antimicrobial peptides, and neutrophil-targeted toxin production (Thammavongsa et al., 2015)*.* The role of specific cytokines and signaling pathways in *S. aureus* infection and its resolution has also been extensively studied in both patients and mouse models (Cho et al., 2010; Feuerstein et al., 2017; Miller and Cho, 2011; Montgomery et al., 2015)*.* Patients with MyD88 or interleukin-1 receptor-associated kinase 4 (IRAK4) deficiencies experience recurrent *S. aureus* infections, which underscores the critical role of TLR and IL1R recognition by innate immune cells in generating an effective inflammatory response against the bacterium (Picard et al., 2010). Mϕs play a particularly important role, as they completely fail to respond to whole streptococci and staphylococci in MyD88/IRAK4 deficiency, while circulating monocytes still mount a residual inflammatory response (Craig-Mueller et al., 2020). In addition, MyD88 in Mϕ has been shown to be critical for abscess resolution in staphylococcal skin infection (Feuerstein et al., 2015). In this context, IL-1β-IL1R signaling has been demonstrated to be critical not only for bacterial clearance but also for wound healing. In staphylococcal skin infection, neutrophils are a primary source of IL-1β, and IL-1β deficiency impairs bacterial clearing and abscess formation (Cho et al., 2012)*. S. aureus* sensing leading to IL-1β production and signaling through MyD88 in keratinocytes has also been shown to boost skin regeneration after injury (Wang et al., 2021).

Another cytokine that is crucial for protection against *S. aureus* skin infections is IL-17A. Its importance is evident in patients with hyper-IgE syndrome (HIES), resulting from mutations in STAT3. Affected humans experience recurrent *S. aureus* skin infections due to a defective Th17 response (Miller and Cho, 2011). Although neutrophils from HIES patients appear to be functional in chemotaxis and ROS production, they are more sensitive to lytic cell death in *S. aureus* infection and have a shorter half-life (Farmand et al., 2018). Moreover, mice deficient in IL-17 signaling spontaneously develop *S. aureus* infections (Cho et al., 2010). In addition, *S. aureus* skin infections induce local hypoxia, leading to induction of the hypoxia-inducible factor 1 a (HIF-1α) and ultimately IL-1β production, both in keratinocytes (Wickersham et al., 2017) and dermal Mϕ (Forde et al., 2023). Mechanistically, Mϕ increase levels of the TCA metabolite succinate under inflammation, which stabilizes HIF-1α (Tannahill et al., 2013). HIF-1α, in turn, induces expression of glycolytic genes, which promote a switch from oxidative phosphorylation to glycolysis and the upregulation of Il-1β in Mϕ (Woods et al., 2022).

Interestingly, however, at 1 day post-infection (dpi), dermal Mϕ can bypass the need for HIF-1α by initiating a metabolic shift and generating an effective inflammatory response in a GM-CSF-dependent manner (Forde et al., 2023). GM-CSF in the steady state is generally considered nonessential for most tissue Mϕ, with the exception of alveolar Mϕ (Guilliams et al., 2013; Kopf et al., 2015). However, in the infected dermis at 1 dpi, γδ T-cells produce GM-CSF, triggering metabolic rewiring in both resident and newly recruited Mϕ. GM-CSF also induces the production of itaconate, a metabolite that is critical for the timely resolution of infection (Forde et al., 2023).

### Trained immunity by skin macrophages and HSCs

Mice previously infected subcutaneously with methicillin-resistant *S. aureus* exhibit increased efficiency in early bacterial clearance in a subsequent infection, independently of T- and B- cells (Chan et al., 2017). In this model, skin lesions of primed mice showed increased neutrophils, Mϕ, Langerin-positive DCs, and NK cells compared to naive controls, as well as elevated IL-17A, IL-22, and IFN-γ levels. Notably, in *Rag1^-/-^* mice, which lack functional T- and B- cells, IL-22 levels were higher compared to naïve mice, confirming an innate source (Chan et al., 2017). In a follow-up study, elevated IL-17, IL-6, CXCL9, and CCL5 levels were observed in the skin after re-infection. The involvement of Mϕ in the training effects was proposed, based on the observed increase in what the authors refer to as M1 Mϕ in the skin of primed mice (Chan et al., 2018). Bone marrow-derived Mϕ (BMDM) of previously infected mice displayed enhanced *S. aureus* killing in vitro and conferred protection when adoptively transferred into a flank of naïve mice. As the effect persisted even after in vitro differentiation of BMDM for two weeks, these findings pointed to hematopoietic stem cells (HSCs) undergoing immune training in *S. aureus* infection, leading to a long-term memory phenomenon (Chan et al., 2018).

Indeed, pivotal studies revealed that β-glucan administration or BCG vaccination can train HSCs in the bone marrow, which enhances myelopoiesis and induces the generation of trained progeny, for example monocytes and Mϕ, and thus improves antimycobacterial immunity (Bekkering et al., 2014; Cirovic et al., 2020; Kaufmann et al., 2018; Mitroulis et al., 2018). Considering that a subcutaneous *S. aureus* infection can cause bacterial spread to liver, spleen, and kidney (Chan et al., 2017), it is conceivable that *S. aureus* also reached the bone marrow in the studies discussed above, inducing memory responses on HSC level, similar to what has been observed with intravenous or subcutaneous BCG vaccination (Kaufmann et al., 2018), although this was not directly assessed.

**Figure 2.**
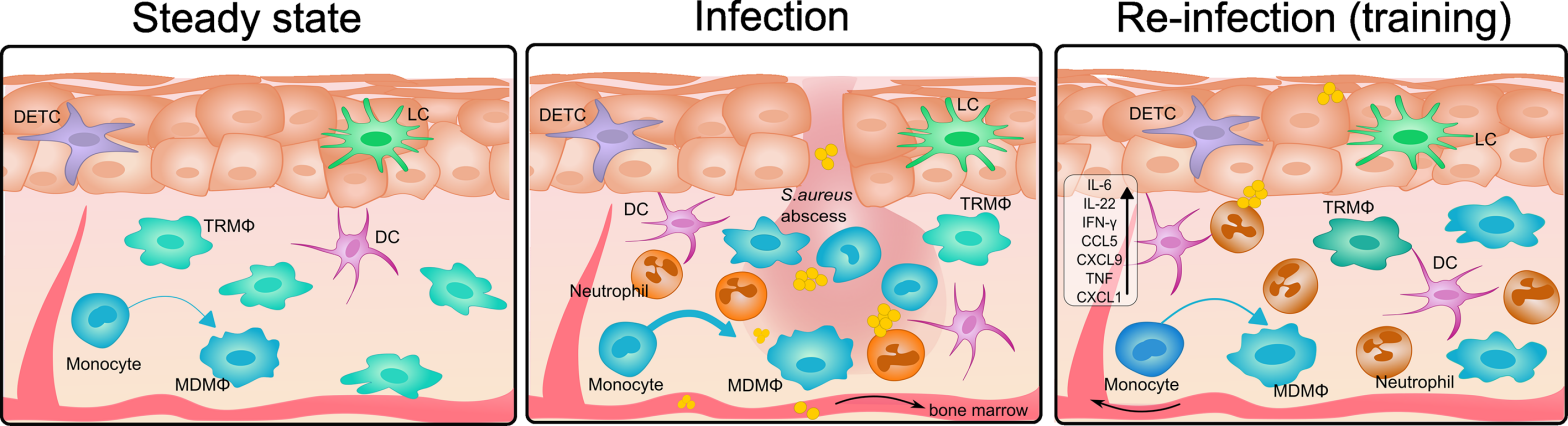
Innate memory formation in *S. aureus* skin infection. In homeostatic mouse skin, tissue resident (TRMΦ) represent the largest innate immune cell population. Monocytes differentiate into macrophages (MDMΦ) over time. Some immune cells of the skin are not represented in this scheme, for example CD4 T cells and γδ T cells. During *S. aureus* skin infection, neutrophils and monocytes rapidly migrate to the site of infection, where abscess formation typically occurs. In some cases, *S. aureus* can disseminate into the circulation, leading to the reprogramming of HSCs in the bone marrow. Tissue-resident MΦ (TRMΦ) at the infection site often undergo necrosis, while infiltrating monocytes differentiate into macrophages (MDMΦ) at an accelerated rate to replenish the population. Upon reinfection, there is an enhanced recruitment of neutrophils, MΦ, and DCs compared to naïve controls, accompanied by elevated levels of inflammatory cytokines and chemokines, including IL-22, CXCL9, CXCL1, TNF, and IFN-γ. The trained phenotype in tissue-resident MΦ wanes over time, as they get gradually replaced by incoming, non-trained monocytes, unless the central innate memory is established, which involves the epigenetic programming of monocyte progenitors in the bone marrow.

In contrast, a strictly localized intradermal *S. aureus* infection, which avoids systemic dissemination, induced a local TI response mediated by the resident dermal Mϕ (Feuerstein et al., 2020). This protection is independent of T-, B-, and NK-cells and is associated with upregulation of STAT1 and CXCL9 in *bona fide* resident dermal Mϕ. Furthermore, although β-glucan-initiated immune memory has been shown to cause histone trimethylation at H3K4 engaging promoter sites of *Myd88* in monocytes (Quintin et al., 2012), TI in dermal Mϕ during *S. aureus* infection appears to be at least partially independent of MyD88 signaling (Feuerstein et al., 2020). Importantly, prolonged GM-CSF production after primary staphylococcal infection is required for improved clearance of a secondary infection and thus for the acquisition of TI (Forde et al., 2023). Next to the GM-CSF phenotype, Mϕ also exhibit a type I interferon (IFN) signature in Mϕ at the stage when *S. aureus* infection is largely cleared (Forde et al., 2023). Interferons (IFNs), both type I and type II, have been identified as important for the development of TI in bone marrow stem cells (Kaufmann et al., 2018) and Mϕ in the lung (Zahalka et al., 2022). A possible role for IFNs in the dermal Mϕ memory response therefore remains to be investigated. However, dermal Mϕ have a relatively short lifespan and high turnover during infection (Figure 2; Forde et al., 2023; Ginhoux et al., 2017; Lohrmann et al., 2025), restricting the duration of the memory response. Indeed, the memory response could be prolonged in *Ccr2^-/-^* mice, in which Mϕ replacement is delayed due to significantly reduced monocyte influx (Feuerstein et al., 2020). These findings suggest that for long-lasting TI in barrier tissues, priming must occur at the HSCs and myeloid precursor level.

**Figure 3.**
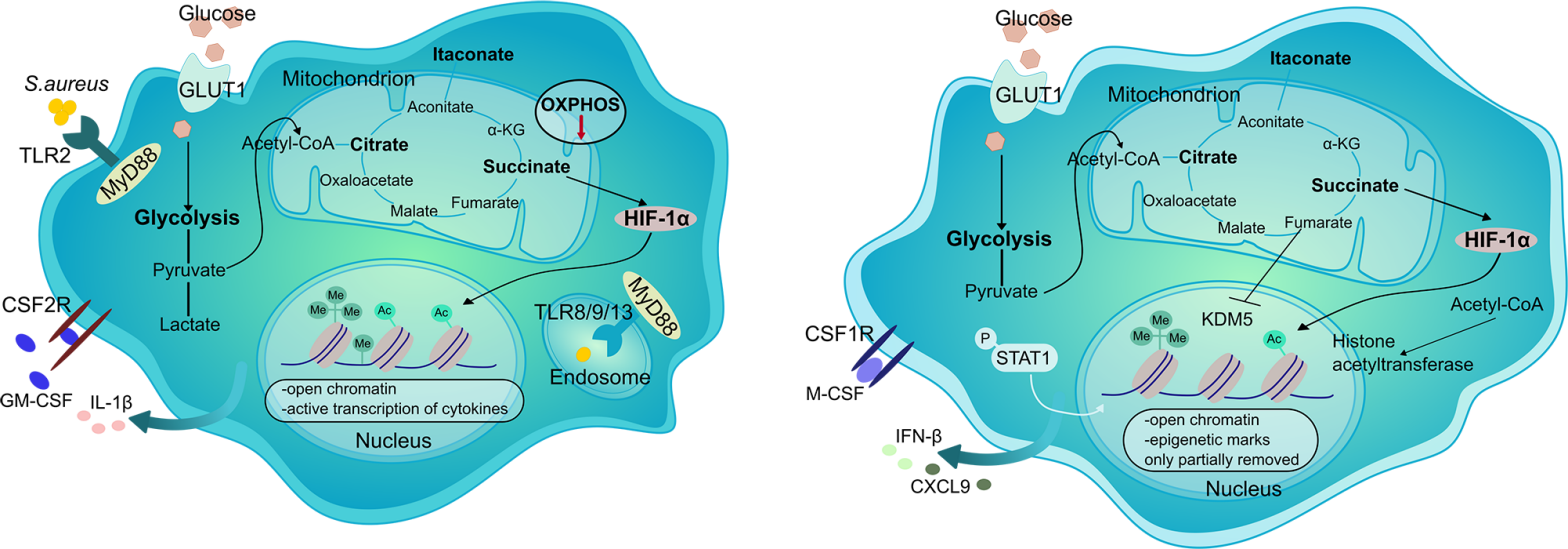
Epigenetic and metabolic reprogramming of dermal MΦ in *S. aureus* infection. MΦ detect *S. aureus* through various pattern recognition receptors (PRRs), such as TLR2 and endosomal TLRs (8, 9, 13). Infection causes a reduction in OXPHOS and an increase in glycolysis, leading to the accumulation of lactate and certain TCA cycle metabolites (e.g., itaconate, succinate). Succinate stabilizes HIF-1α, which then triggers the expression of glycolytic genes early in the infection. GM-CSF signaling bypasses the reliance on HIF-1α. DNA methylation and chromatin unfolding enable rapid transcription of inflammatory mediators. Once the infection is cleared, some of the macrophage alterations remain, allowing for an altered immune response during subsequent infections.

Metabolic changes are intertwined with the development and maintenance of epigenetic changes that control innate memory (Figure 3). Glycolysis has been shown to be essential for TI in monocytes (Cheng et al., 2014), and polymorphisms in glycolytic genes (e.g. HK, PFKP) reduce the production of TNF and IL-6 after re-stimulation (Arts et al., 2018a). In trained monocytes, glycolysis feeds the pentose phosphate pathway (PPP), which supports the production of nucleotides and NADPH (Arts et al., 2016). In addition, immune training is attenuated when cholesterol synthesis, fatty acid oxidation, or glutaminolysis are blocked (Arts et al., 2016; Zahalka et al., 2022). As an example, glutaminolysis leads to an accumulation of α-ketoglutarate and fumarate, which enhances epigenetic memory by inhibiting KDM5 demethylases responsible for demethylating histone H3K4 (Arts et al., 2016). In principle, histone modifications are rapidly induced by an inflammatory stimulus and are only partially reversed when the cell returns to a resting state. This residual marking facilitates chromatin accessibility and therefore faster transcription of inflammatory mediators in response to subsequent challenges. Inhibition of demethylases and deacetylases or activation of lysine methyl-transferase by metabolites therefore directly connects metabolic and epigenetic reprogramming in innate memory (Bekkering et al., 2014; Kleinnijenhuis et al., 2012; Mitroulis et al., 2018).

In the context of skin infection, the pathogen itself could modulate the immune response and induction of TI by the host. For example, *S. aureus*-derived lactate inhibits the histone deacetylase 11 (HDAC11), leading to upregulated HDAC6 activity and increased acetylation of histone 3 at the Il-10 promoter in Mϕ (Heim et al., 2020). The importance of host-pathogen interactions and competition for nutrients was illustrated using the ΔhemB *S. aureus* strain, which relies solely on glycolysis. Re-infection with the ΔhemB strain led to local fumarate reduction in the skin and ultimately inhibited the development of TI (Wong Fok Lung et al., 2020). Moreover, specific *S. aureus* variants isolated from the skin of chronically colonized patients had polymorphisms in genes associated with enhanced glycolysis and limitation of fumarate generation, and one of these strains led to a decrease in innate memory as compared to the prevalent USA300 strain (Acker et al., 2019). Interestingly, a recent study revealed that although mice exposed to *S. aureus* or heat-fixed *C. albicans* develop trained immunity, enhanced immune responses paradoxically boost persistence of antibiotic-resistant *S. aureus* (Lin et al., 2024). This phenomenon was specifically attributed to the accumulation of sodium fumarate (independently of other fumarate derivatives) and could be reversed by the administration of an antidiabetic drug metformin, which acts as an mTOR inhibitor and trained immunity suppressor (Lin et al., 2024).

## Trained immunity in the skin beyond macrophages

### Granulocytes

In contrast to Mϕ, granulocytes are short-lived, thus their TI mechanisms are likely different. A recent study demonstrated that epicutaneous *S. aureus* infection triggers epigenetic and metabolic reprograming of the eosinophil progenitors in the bone marrow, leading to an enhanced subsequent allergic sensitization both at the site of infection (skin) and also in a distal site (lung) (Radhouani et al., 2025). This effect was transferable by bone marrow transplantation and was driven by soluble mediators, for example IL-33. In particular, eosinophils lacking the IL-33 receptor exhibited impaired bone marrow expansion post-infection and a reduced lung infiltration upon allergen inhalation (Radhouani et al., 2025).

Similarly, neutrophils appear to acquire memory-like properties primarily through alterations in their bone marrow progenitors, rather than through changes in their terminally differentiated state (Moorlag et al., 2020). After intradermal BCG vaccination of healthy volunteers, neutrophils displayed changes for 90 days post vaccination, such as increased activation markers (CD11b, CD66b), and enhanced antimicrobial functions (degranulation, phagocytosis, ROS production) upon secondary stimulation ex vivo. These changes were associated with sustained epigenetic modifications, such as increased H3K4me3 at promoters of genes involved in inflammation (e.g. STAT4) and metabolism (e.g. mTOR and PFKP; Moorlag et al., 2020).

Lastly, while skin bacterial infection can cause basophil recruitment and IL-4 release, which suppresses local IL-17A-dependent protective responses and may promote persistent changes in the skin immune environment, direct evidence for long-term, systemic TI is still emerging (Leyva-Castillo et al., 2024). The regulatory role of basophils in local inflammation and their potential to modulate other immune cells suggest they could contribute to lasting immune changes after skin infection (Leyva-Castillo et al., 2024). It seems important to note that inherent challenges to determining TI induced by BCG remain. First, the inter-individual and longitudinal variability, for example in the immunophenotype of neutrophils is high. Furthermore, BCG can persist for months in tissue niches like the skin, and potentially the bone marrow, making the distinction between TI and effects associated with chronic infection difficult (Hoft et al., 1999).

### Dendritic cells

Compared to other myeloid cells, the capacity of DCs to develop memory-like features has been less well investigated. This may be due to their well-characterized role in T cell activation, which may blur the distinction between TI and adaptive immunity. For example, Antonio-Herrera and colleagues have shown that an adjuvant derived from cholera toxin (CTB) activates DCs in the skin, promoting long-term maintenance of CD4 T cells, in particular Th17 and Th1 subsets (Antonio-Herrera et al., 2018). In a subsequent study, the same group showed that CTB can directly induce TI in DCs, characterized by higher TNF production, CD86, and lactate dehydrogenase (LDH) expression upon re-stimulation (Tepale-Segura et al., 2024). Importantly, this trained phenotype was observed both locally in the skin and systemically in bone marrow DC precursors (Tepale-Segura et al., 2024). Notably, immune memory of DCs was shown to be dependent on histone methylation, as demonstrated in vitro in DCs isolated from mice immunized using a specific *C. neoformans* strain. In this context, DCs were able to produce higher levels of IL-2, IFN-γ, IL-4, and TNF upon secondary challenge (Hole et al., 2019).

### Natural killer (NK) cells

One of the first reports on TI identified NK cells with a memory phenotype after cytomegalovirus (CMV) infection (Sun et al., 2009). Infection with human CMV (HCMV) promotes the induction and long-term maintenance of expanded NK cell populations. Resulting innate IFN-γ–producing NK cells mediate rapid but short-lived protection (Luetke-Eversloh et al., 2014). In this context, the underlying mechanism of TI is mediated by pro-inflammatory cytokines, which induce demethylation of DNA at loci encoding antiviral cytokines such as IFN-γ. This cytokine-driven epigenetic remodeling of chromatin was seen to enhance the accessibility of these genes, thereby promoting increased IFN-γ production upon subsequent stimulation (Luetke-Eversloh et al., 2014). Similarly, in vitro stimulation of NK cells isolated from BCG-vaccinated mice with *S. aureus* resulted in enhanced and sustained production of pro-inflammatory cytokines, including IFN-γ (Kleinnijenhuis et al., 2014).

Although the aforementioned studies addressed circulating NK cells in systemic infection, the principles may also apply to tissue-resident NK cells, including those in the skin. Although NK cells are not typical constituents of a healthy skin (Torcellan et al., 2024), in conditions like atopic dermatitis, they can home to the skin and initiate a cytotoxic response (de Lima et al., 2024). The bactericidal properties of NK cells rely on HIF-1α, as mice lacking HIF-1α in NKp46+ cells fail to control wound healing and bacterial dissemination in *S. aureus* and group A streptococcal skin infection (Sobecki et al., 2021). Following infection, Torcellan and colleagues elucidated the capacity of NK cells to differentiate into long-lived, resident memory-like cells, with increased immune responses after secondary exposure to either vaccinia virus or *S. aureus*. Thus, differentiated tissue-resident NK (trNK) cells can rapidly transform into effector NK cells, exhibiting an enhanced immune response (Torcellan et al., 2024).

### Immune memory in non-immune cells

Epithelial stem cells (EpSCs) in the basal layer of the epithelium are self-renewing cells maintaining the epithelial tissues of the skin, the gastrointestinal lining, and the respiratory tract (Blanpain et al., 2004).

Naik et al. found that skin inflammation induces long-lasting memory in EpSCs, enabling faster wound closure upon subsequent injury. This process relies on AIM2, as *Aim2*-deficient mice show impaired wound healing post-inflammation (Naik et al., 2017). Following an initial inflammatory event, primed EpSCs showed increased chromatin accessibility of stress-response genes, allowing for rapid transcriptional activation after a secondary challenge. EpCS memory was localized and not transmittable through circulation to naïve sites and was independent of Mϕ, T- and B-cells. The memory of EpSC was sustained for at least 180 days, likely due to their long lifespan and self-renewal capacities (Naik et al., 2017).

Other non-immune cells with possible direct or indirect implication in TI are fibroblasts, which are important for the formation of extracellular matrix (ECM) components, for wound healing and tissue remodeling. As the most abundant stromal cells in the dermis, their primary function is to provide structural tissue support. However, growing evidence suggests that fibroblasts may also contribute to immune memory. For example, in vitiligo, IFNγ-responsive fibroblasts were identified as key players in the recruitment and activation of auto-reactive CD8 +cytotoxic T-cells through the secretion of cytokines, ultimately leading to the loss of melanocytes (Xu et al., 2022).

In the synovium of patients with rheumatoid arthritis, a fibroblast subpopulation has been implicated in disease promotion via the formation of cytokines and matrix metalloproteinases (Mizoguchi et al., 2018). The abnormal inflammatory profile of fibroblasts in these diseases has not yet been linked to the long-term reprogramming associated with TI. However, their longevity and widespread presence in tissues highlight the necessity to further investigate their potential role in this context.

## Skin microbiota and TI

From birth on, the skin must rapidly adapt to exposure by an increasing complex mixture of bacteria, fungi, viruses, and archaea. The mode of delivery has been found to impact the composition of the early-life skin microbiota. While infants born vaginally have *Lactobacillus* as the predominant microbial constituent, those delivered by cesarean section carry more skin-associated bacteria, such as *Staphylococcus* and *Corynebacterium species*, with an overall lower microbial diversity (Dominguez-Bello et al., 2010; Chu et al., 2017). As individuals mature, the skin microbiota undergoes substantial changes, including a decline in *Staphylococcus* and *Streptococcus*, greater site-specific specialization, and an increase in lipophilic organisms, such as *Cutibacterium* and *Corynebacterium spp*. (Luna, 2020; Oh et al., 2012). The microbiota serves as a continuous source of effector molecules (e.g. bacterial DNA and rRNA) that can diffuse across the epidermis to dermis and superficial adipose tissue, engaging pattern recognition receptors (PRRs; Nakatsuji et al., 2013). However, the mechanisms determining whether microbial signals are tolerated, or whether they induce a robust immune response by the host cells, remain incompletely understood and may depend both on host age and the specific commensal species involved. For example, skin colonization with commensal bacteria promoted tolerance and led to accumulation of Tregs in the neonatal period, an effect not recapitulated in adult mice, highlighting the importance of exposure to microbiota early in life (Scharschmidt et al., 2015). Furthermore, microbiota disruption by antibiotic treatment ameliorated psoriasis in adult mice, but led to disease exacerbation in neonatal mice (Zanvit et al., 2015).

In contrast, certain commensal species can promote inflammation. *Cutibacterium acnes* colonizes the lipid-rich, hypoxic environment of the hair follicle where it produces short-chain fatty acids (SCFAs), which act as histone deacetylase inhibitors in keratinocytes (Sanford et al., 2016). This epigenetic change can shift keratinocytes from a tolerogenic to an inflammatory phenotype, illustrating a direct effect of microbial metabolites on immune response modulation. Similarly, colonization by *Staphylococcus epidermidis*, one of the most abundant commensal species in the skin, can activate dermal DCs and promote IL-17A-mediated T cell responses (Naik et al., 2015).

While it is established that microbial products can promote or inhibit histone modifications, their long-term effect on TI has yet to be directly demonstrated. There is, however, evidence that the interplay between microbiota and TI might be bi-directional. For instance, BCG vaccination has been shown to induce memory in alveolar Mϕ independently of circulating monocytes, but also to alter the intestinal microbiome and systemic metabolites (Jeyanathan et al., 2022). Moreover, the microbiota plays a critical role in myelopoiesis, as germ-free and antibiotic-treated mice have reduced numbers of myeloid cell progenitors in bone marrow and other myelopoietic defects (Khosravi et al., 2014). These findings indicate the possibility that microbiota-derived metabolites may induce TI by acting locally at the skin barrier, or systemically through effects on bone marrow progenitors. However, this hypothesis has yet to be experimentally validated.

## Trained immunity in other skin pathologies

Recent discoveries in TI mechanisms have not only offered insights into previously unexplained disease causes and consequences but also have opened new therapeutic avenues. Most prominently, BCG vaccination induces TI, suggesting a mechanistic explanation for the long-established ‘off-target’ effects in non-mycobacterial diseases where a strong immune response may be beneficial, in particular in cancer. BCG has been used for a long time as a treatment for melanoma by direct intradermal or subcutaneous administration into metastatic lesions (Morton et al., 1974). This approach has been shown to convert the tolerogenic tumor environment to a more inflammatory one, characterized by an immune cell influx and consequential elevated inflammatory cytokine profile. Accordingly, tumor-associated Mϕ are a major contributor to the melanoma environment, with their inflammatory phenotype being linked to a better prognosis. Beyond the immediate immune response, the potential long-term effects of BCG-induced TI are important to consider. Notably, BCG vaccination has been observed to reduce the risk of melanoma development (Pfahlberg et al., 2002), suggesting that TI may poise Mϕ for a faster response and immune surveillance against tumor formation.

On the other hand, the enhanced inflammatory response mediated by innate immune memory might contribute to the disease pathology. Indeed, monocytes from patients suffering from different autoimmune and autoinflammatory diseases show a phenotype resembling innate immune memory that is increased cytokine production, as well as metabolic and epigenetic rewiring (Arts et al., 2018b). In inflammatory skin conditions, the cross-talk of epithelial and IL-17 producing immune cells is critical for disease progression. Namely, metabolic rewiring of highly glycolytic, lactate-producing epithelial cells has been shown to enhance the IL-17 production by γδ T-cells (Subudhi et al., 2024). Inhibition of HIF-1α alleviated the inflammatory symptoms. This observation ties into previous findings on trained monocytes being characterized by high glucose consumption, lactate production, and mTOR activation through HIF-1α (Cheng et al., 2014). TI may also be linked to disease progression in the autoimmune disorder vitiligo, in which autoreactive cytotoxic T-cells attack melanocytes in the epidermis (Post et al., 2023). In patients with vitiligo, an enhanced inflammatory response was found both in circulating monocytes and in structural cells in the skin, for example keratinocytes (Li et al., 2020) or fibroblasts (Xu et al., 2022).

In conclusion, the growing mechanistic understanding of TI offers important diagnostic and therapeutic opportunities for a number of chronic inflammatory and oncologic diseases.

## Summary and conclusion

Starting at birth, the integration of genetic programs underlying tissue growth and development and exogenous cues from the emerging microflora shapes skin immunity both at the cellular and functional level. Heterocellular interactions and a dynamic interplay of resident and incoming immune cells tune tissue immune cell composition and activity and modulate the response to subsequent infections, that is local TI. In TI development, cellular metabolites, for example itaconate and fumarate are involved, as are epigenetic modifications.

It is an attractive scenario that signals derived from epithelial cells and further long-lived structural cells such as fibroblasts or nerves may lead to an enduring modulation of secondary innate responses. Moreover, the influence of environmental factors beyond those of microbial origin, such as UV light or mechanical insults on TI in the skin, needs to be disentangled. For instance, UV light is a well-recognized modulator of innate immune cells in the skin. UV radiation induces DNA damage, leading to PRR activation and production of type I interferons, antimicrobial peptides, and cytokines (Tang et al., 2024). However, the UV radiation effects are highly dependent on exposure intensity, duration, and a specific UV light wavelength (Bernard et al., 2019). Notably, excessive or chronic UV exposure has an immunosuppressive effect and is associated with higher skin cancer risk (Becklund et al., 2010). Nonetheless, a direct connection between exposure to UV radiation and the induction of TI in the skin is yet to be investigated (Tang et al., 2024).

Finally, TI may even profoundly affect general features of the skin including immune cell adaptation, tissue turnover and repair, and prevention of early tumor growth. It is an intriguing outlook that expanding knowledge in these areas may eventually allow us to harness TI for preventing and treating pathological skin conditions in the future.
